# Clinical Uses of Electricity

**Published:** 1874-03

**Authors:** 


LECTURES ON THE CLINICAL USES OF ELECTRICITY. DELIVERED IN UNIVERSITY COLLEGE HOSPITAL. *By J. Russell Reynolds, M.D., F.R.S., Professor of the Principles and Practice of Medicine, in University College, etc., etc. Second Edition. Philadelphia: Lindsay & Blakiston,* 1874. 12 *mo. Cloth, pp.* 118. *Price,* $1.25.

